# Supplementary material for: Spatially inhomogeneous competition between superconductivity and the charge density wave in YBa2Cu3O6.67
Source: Nat Commun. 2020 Feb 20;11:990. doi: 10.1038/s41467-020-14536-1 (PMC7033133; doi:10.1038/s41467-020-14536-1)
Supplement: Supplementary file 1 — Supplementary Information [file 41467_2020_14536_MOESM1_ESM.pdf]

Supplementary Information

Spatially Inhomogeneous Competition between Superconductivity and the Charge Density Wave in $YBa_2Cu_3O_{6.67}$

J. Choi *et al*.

I. SUPPLEMENTARY INFORMATION

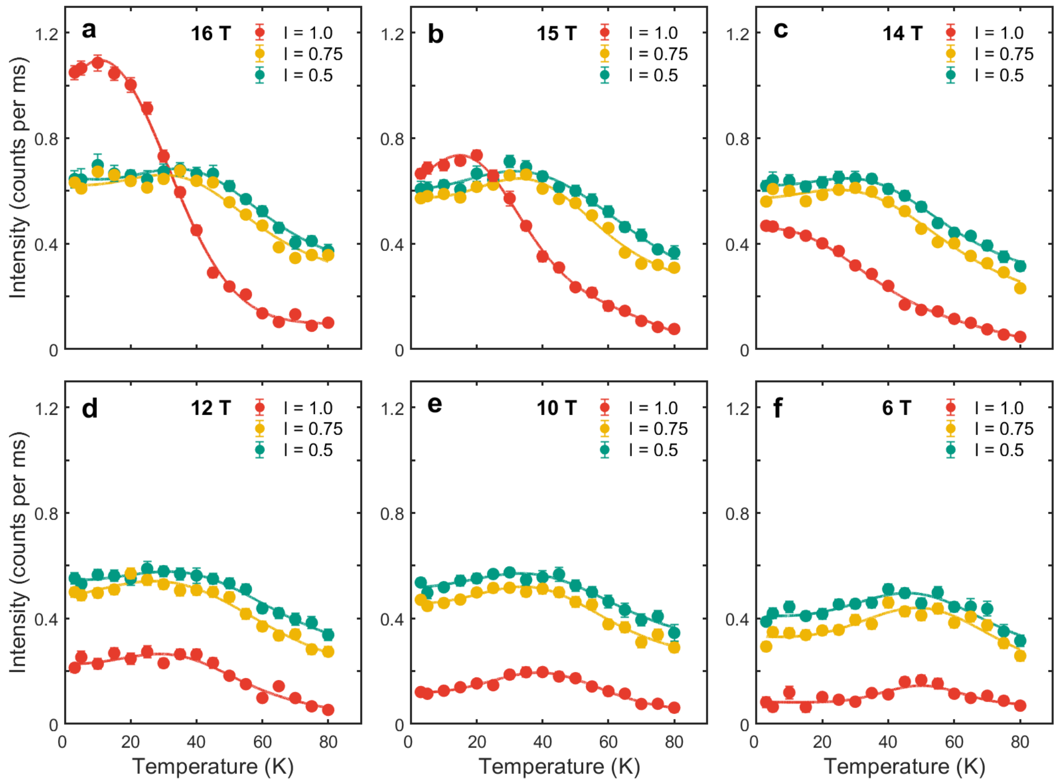

Supplementary Figure 1. **Temperature dependence of diffraction intensity at different ℓ positions.** Diffraction intensity measured at $\ell = 0.5$ (green), 0.75 (yellow), and 1 (red) are plotted for comparison as a function of temperature at different magnetic fields as indicated: (a) 16 T (b) 15 T (c) 14 T (d) 12 T (e) 10 T and (f) 6 T. Here, $\ell = 0.5$ and $\ell = 1$ data are taken from 5b and 5c, respectively. For $\ell = 0.75$, the diffracted intensity is integrated and averaged over a range of $0.65 < \ell < 0.85$ from ℓ-scans after subtraction of linear backgrounds. Source data are provided as a Source Data file.

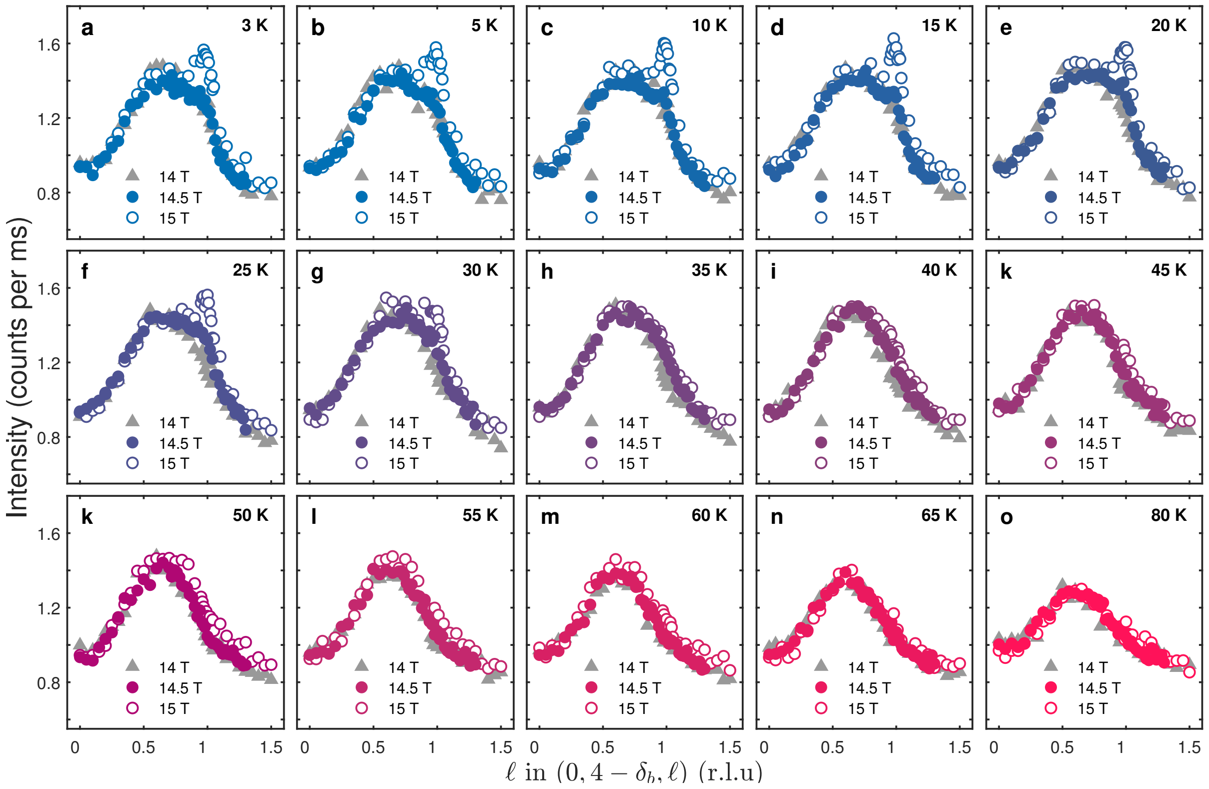

Supplementary Figure 2. **Out-of-plane scans (ℓ-scans) through** $(0, 4-\delta_b$**,1). (a-o)** Raw ℓ-dependent intensity profiles are plotted at various temperatures as indicated. The data measured at $B = 14$ T (grey triangles), 14.5 T (solid circles) and 15 T (open circles) are superimposed at each temperature. Source data are provided as a Source Data file.

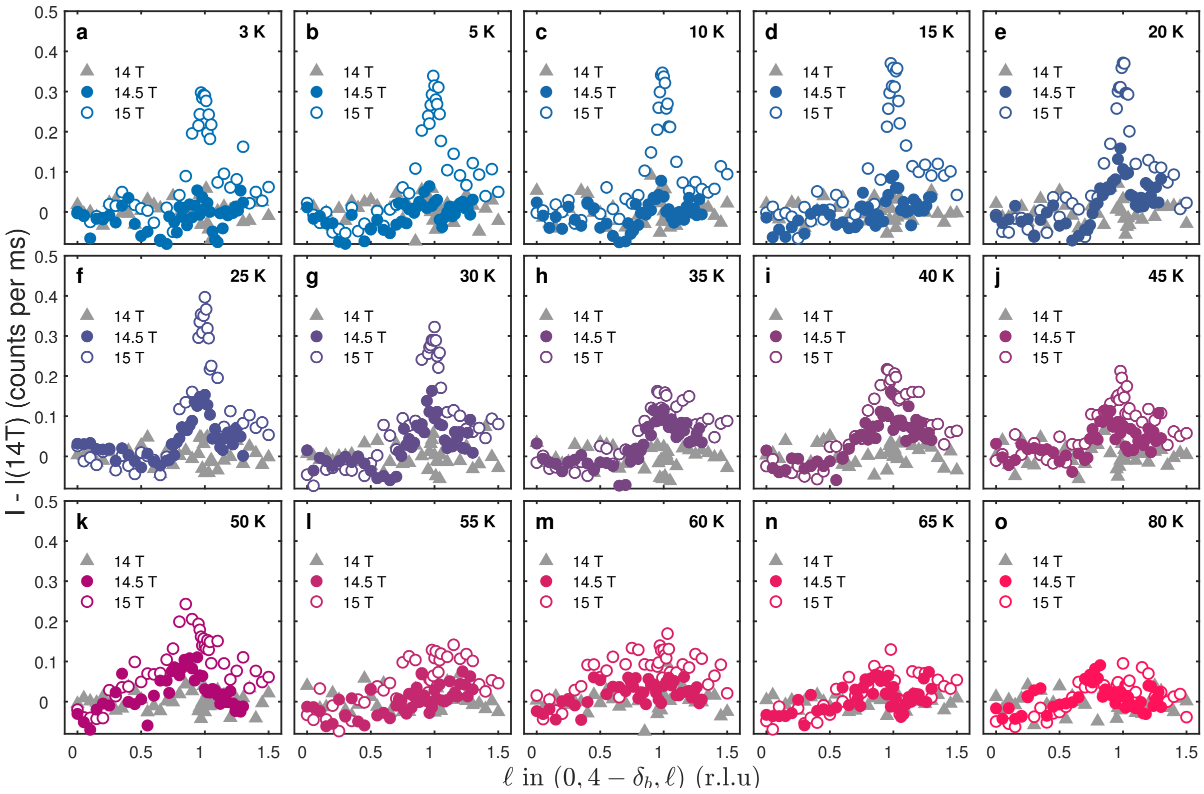

Supplementary Figure 3. **Background subtracted** ℓ**-scans through** $(0, 4 - \delta_b,$**1). (a-o)** background subtracted ℓ-dependent intensity profiles are plotted at various temperatures as indicated. Gaussian fits of 14-T data are used as the backgrounds at each temperature. Source data are provided as a Source Data file.

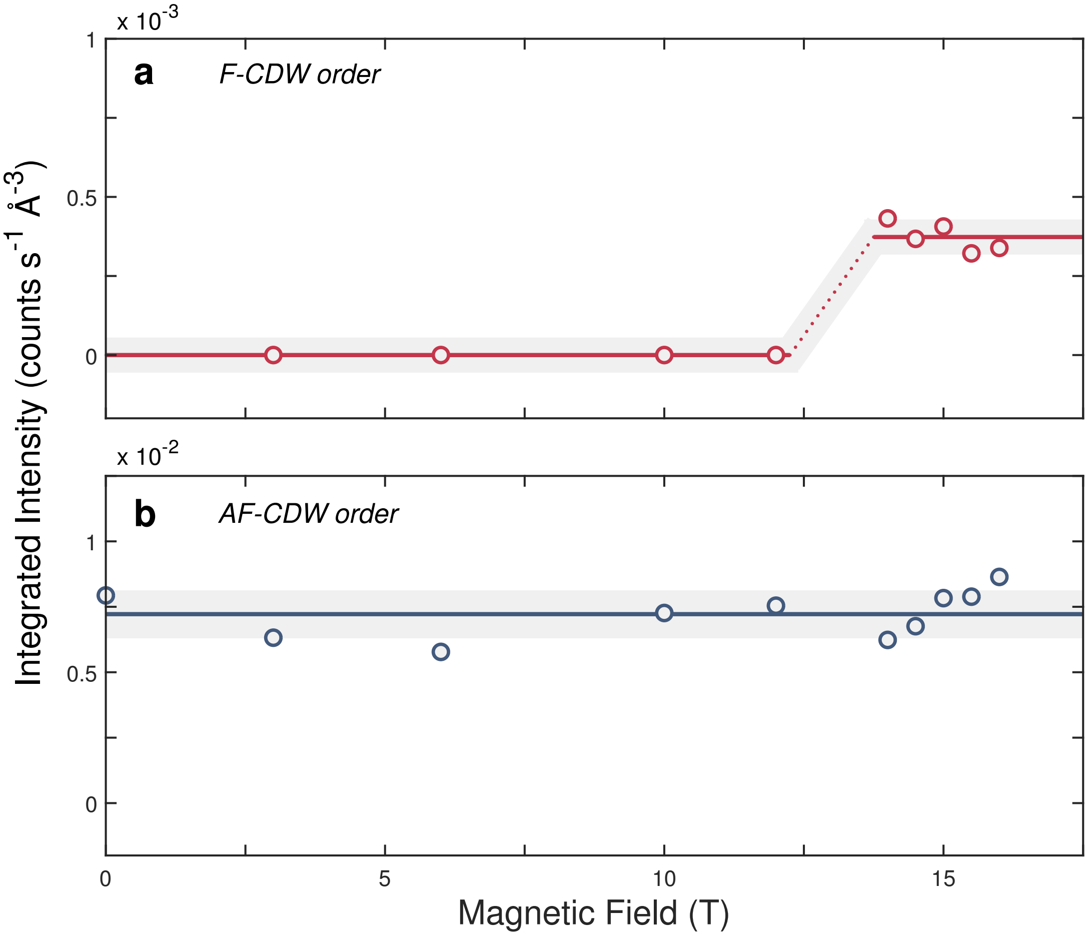

Supplementary Figure 4. **Integrated intensity of charge density wave order reflections.** Intensity of **(a)** ferro and **(b)** antiferro-coupled charge density wave (CDW) reflections integrated in the three-dimensional reciprocal space, calculated by $I_{\mathrm{CDW}} \times \xi_b^{-2} \times \xi_c^{-1}$, is plotted against a magnetic field. Here, I_{CDW} is the peak intensity at $(0, 4-\delta, 1/2)$ (AF-CDW) and $(0, 4-\delta, 1)$ (F-CDW) measured at 3 K. ξ_b and ξ_c are the correlation lengths of CDWs along the crystallographic b- and c-axis direction at 22 and 3 K, respectively. Due to the instrumental constraint, we have limited information about the CDW correlation lengths in the a-axis direction, but they appear to be similar to those along the b-axis. We have therefore used ξ_b^2 as a substitute for $\xi_a \times \xi_b$ to estimate the reciprocal-space integrated intensity. Source data are provided as a Source Data file.
